# Associations between use of renovated urban parks and perceptions of social cohesion in diverse New York City communities

**DOI:** 10.1186/s12982-026-01475-3

**Published:** 2026-02-10

**Authors:** Justine Maffei, Rachel L. Thompson, Katarzyna E. Wyka, Emma Tsui, Nevin Cohen, Nasim Sabounchi, Terry T.-K. Huang

**Affiliations:** 1 Center for Systems and Community Design and NYU-CUNY Prevention Research Center, Graduate School of Public Health and Health Policy, City University of New York, New York, NY USA; 2 Department of Epidemiology and Biostatistics, Graduate School of Public Health and Health Policy, City University of New York, New York, NY USA; 3 Department of Community Health and Social Sciences, Graduate School of Public Health and Health Policy, City University of New York, New York, NY USA; 4 Department of Health Policy and Management, Graduate School of Public Health and Health Policy, City University of New York, New York, NY USA

**Keywords:** Parks, Built environment, Social cohesion, Sense of community, Health equity, Urban health

## Abstract

Social cohesion supports urban community functioning and mental and physical health. High-quality neighborhood green spaces have the potential to improve social cohesion by fostering social connections among community members. The Community Parks Initiative (CPI) is an equity-focused initiative that led to the redesign and renovation of urban parks in diverse New York City neighborhoods with a history of disinvestment. We analyzed cross-sectional, population-representative survey data (*n* = 2,000) from eight neighborhoods with recently renovated CPI parks to assess the relationship between self-reported renovated park use frequency in the past month and perceived neighborhood social cohesion, measured by the Social Cohesion and Trust (SCT) subscale of the Collective Efficacy Scale and the Sense of Community Index (SCI-2). Linear regression models showed that each additional CPI-renovated park visit in the past month was associated with higher perceived social cohesion and trust (SCT β = 0.012, 95% CI: 0.008–0.016) as well as higher perceived sense of community (SCI-2 β = 0.464, 95% CI: 0.335–0.593) after adjusting for sex, age, race/ethnicity, income, employment, education, use of non-CPI parks, and park site. Stratified analyses showed the strongest associations among individuals with annual household incomes of $75,000–$150,000 (SCI-2 β = 0.854, 95% CI: 0.491–1.217) and among Non-Latino/a Black (SCI-2 β = 0.478, 95% CI: 0.243–0.714) and Latino/a individuals (SCI-2 β = 0.676, 95% CI: 0.480–0.871). These findings highlight the potential of high-quality urban green spaces in promoting positive perceptions of community social cohesion, particularly in middle-income and minority communities.

## Background

Social cohesion, broadly defined as the connectedness and solidarity among members of a community [1], is an important but under-appreciated determinant of health in modern society [2]. Although definitions vary [3, 4], most emphasize two fundamental components: a sense of belonging and relationships between community members [1]. Empirical evidence at both the individual and community level indicates that higher levels of social cohesion may be associated with better mental health [5–7] and cardiovascular outcomes [8–11]. For example, a recent review of 13 prospective longitudinal studies found that community social cohesion was associated with fewer depressive symptoms among adolescents and young adults [6]. Similarly, a large, nationally-representative study of adults in the United States (U.S.) found that low perceived community social cohesion was associated with a 75% higher prevalence of severe psychological distress [7]. Among a nationally-representative sample of U.S. adults aged 50 years and older in the Health and Retirement Study cohort, each standard deviation increase in perceived neighborhood social cohesion was associated with 22% lower odds of myocardial infarction [8] and 15% lower odds of stroke [9] over a four-year prospective follow-up period.

Social cohesion not only has the potential to support one’s health and wellbeing but is also an important component of the socio-ecological functioning of an urban community [4]. Communities may boost social cohesion by providing their members access to public amenities with the potential to improve inter-group relationships and foster social interaction [4]. One such amenity is public parks, which qualitatively provide individuals with a physical connection to nature as well as social connections to friends, family, and the larger community [12]. Public parks can serve as a comfortable setting for repeated visual contacts and short outdoor conversations, which may strengthen relationships between community members [13]. Studies have also shown that simply being in the passive presence of community members while engaging in solitary activities, such as walking, has the potential to strengthen social ties among neighbors [14].

High-quality urban green spaces may play an especially important role in fostering social cohesion and promoting health in disadvantaged minority communities [15]. These communities often bear disproportionate burdens of poor health and fragmented social networks due to systemic factors like housing instability and disinvestment in communal public spaces [16–19]. As a result, low-income urban neighborhoods with predominantly minority populations frequently have limited access to parks and green spaces, which tend to be lower in quality, characterized by fewer amenities, inadequate maintenance, and safety concerns [20–22]. At the same time, research suggests that Black, Latino/a, and Asian individuals tend to use parks for more social, community-oriented activities compared to their White counterparts [23]. For example, among park users in Chicago, White individuals were more likely to visit the park alone, or accompanied by one other person, while minority individuals were more likely to visit with their families or a larger social group [24]. Large-scale investments in park quality improvement may therefore offer a promising strategy for strengthening community social cohesion among racial and ethnic minority groups. However, few studies have explored associations between frequency of park use (irrespective of park quality) and perceived community social cohesion, and no studies to-date have examined the extent to which use of high-quality urban green spaces exclusively may contribute to community social cohesion among diverse communities.

**Fig. 1:**
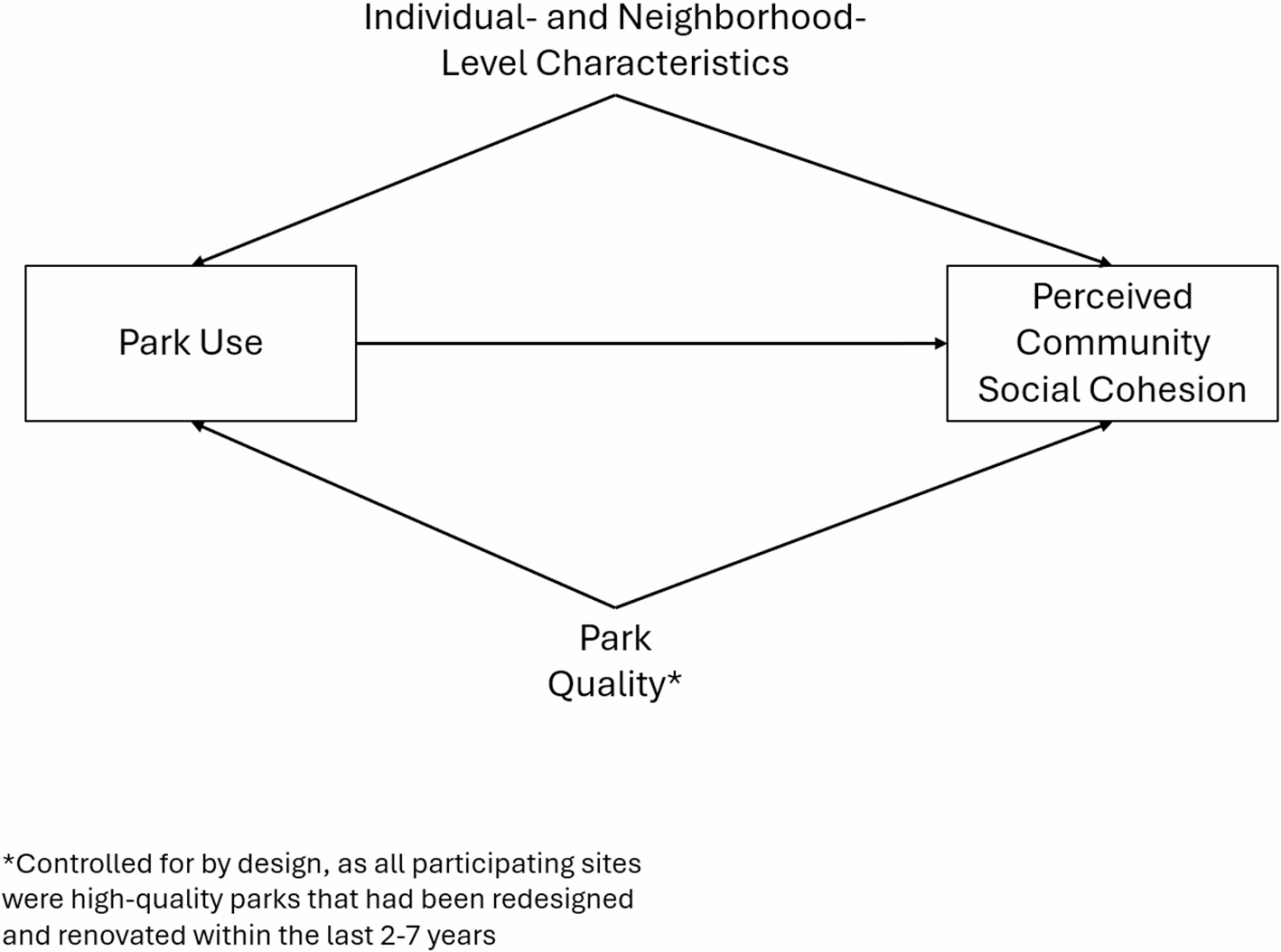
Conceptual diagram with hypothesized relationships between park use, park quality, and perceived community social cohesion

The Community Parks Initiative (CPI) is an ongoing equity-based urban park redesign and renovation program taking place in low-income, diverse neighborhoods in New York City (NYC). To date, dozens of parks have been renovated as a part of CPI, receiving updates such as beautification, seating and shading, adult fitness equipment, ball courts, playgrounds, and expanded green space [25]. This initiative has provided us with a novel opportunity to examine associations between park use and community social cohesion *using data drawn exclusively from neighborhoods surrounding high-quality*,* recently renovated parks*. This is an analytically important consideration, as low-quality parks have the potential to threaten social cohesion if they are perceived as unsafe or disorderly [26]. In other words, the focus on renovated parks allows us to control for important differences in park quality that might otherwise confound the association between park use and social cohesion (Fig. 1). To this end, we investigated the relationship between park use frequency and perceptions of social cohesion in a large, population-representative sample of individuals living near eight recently renovated CPI parks situated in low-income NYC neighborhoods.

## Methods

### Setting

The Supporting Parks and Revitalizing Communities Study (SPARCS) is an ongoing community engagement intervention study across eight newly renovated CPI sites in NYC, launched in June 2023 [27]. Study parks were selected based on previous CPI renovation, adequate population size (*n* ≥ 20,000) and pre-existing community partnerships, with two sites located in each of Manhattan, Brooklyn, Queens, and the Bronx. The eight sites included in this study received substantial quality improvements through comprehensive redesign and renovation as a part of the CPI. Renovations for the eight sites were completed between June 2016 and April 2021 with a median capital investment of U.S. $5.2 million [28].

### Participants and data collection

The analyses reported in this paper utilized cross-sectional data from baseline surveys administered via the SPARCS study. Recruitment and survey data collection strategies for the study have been previously described [29]. Briefly, study participants were recruited from the population living within a 0.5-mile radius of the eight recently renovated CPI park locations by a polling firm (Consensus Strategies, Waltham, MA). Participants were contacted via email or text message. The final sample was obtained through stratified random sampling, with a minimum of 25 participants in each stratum. Strata were defined using sex, age, race/ethnicity, and education level, with enrollment targets chosen to reflect the true demographic distribution of the general population within each neighborhood in the study. A total of 2,067 participants responded to the survey in June 2023. The survey was administered in English, Spanish, and Chinese. Raked weights were applied to survey responses from each of the eight study sites to achieve a population-representative sample, resulting in a final *n* = 2,000 (*n* = 250 per study site).

### Measures

#### Exposure: park use frequency

Park use frequency was assessed with the question: “In the past 30 days, on average, how often have you visited [STUDY PARK NAME] ?”, which has demonstrated excellent test-retest reliability (intra-class correlation > 0.8) in previous validation studies [30, 31]. Response options included: *“Have not visited the park in the last 30 days*,*” “Less than once per month”*,* “Once per month*,*” “2–3 times per month*,*” “Once per week*,*” “2–3 times per week*,*” “4–6 times per week*,*”* and *“Daily.”* The eight-category variable was transformed into a continuous variable by converting the values to the number of visits per month based on the category’s midpoint, as described in a previously published protocol [32]. For example, those who reported daily park use were assigned a value of 30, those who visited 2–3 times per week were assigned a value of 10, and those who visited once per week were assigned a value of 4. In addition, a dichotomous variable was created using the original responses: *“< Once per week”* and *“≥ Once per week”.* Both the continuous and dichotomous park use measures were separately analyzed as exposures to measure associations between park use frequency and perceptions of community social cohesion. The continuous variable captured the full range of park use frequency, allowing for dose-response modeling, while the dichotomous variable was created to distinguish between infrequent and frequent park users, aligning with prior research demonstrating health benefits associated with weekly park use [29, 33–36].

#### Outcomes: social cohesion and trust and sense of community

Social cohesion and trust was assessed using the five-item Social Cohesion and Trust (SCT) subscale of the Collective Efficacy Scale, which measures how likely neighbors are to support one another in times of need on a five-point Likert scale [37]. A continuous scale was constructed after reverse-coding positively worded items and calculating a score for each respondent by averaging their ratings across all five items. The SCT score was analyzed as a continuous outcome ranging from 1 to 5, where higher values indicated greater perceived social cohesion and trust.

In addition to SCT, we also used the Sense of Community Index v.2 (SCI-2), a validated scale used to quantify an individual’s perception of their own community through the following elements: membership, influence, meeting needs, and a shared emotional connection [38, 39]. The scale is composed of 24 questions, each measured using a 4-point Likert scale. Responses were summed and analyzed as a continuous outcome ranging from 0 to 72.

Compared to the SCT scale, the SCI-2 offers a more comprehensive assessment of perceived community social cohesion. We included both measures because they capture complementary dimensions of community social cohesion: SCT focuses on interpersonal trust and mutual support among neighbors, while SCI-2 reflects a broader psychological sense of belonging, influence, and emotional connection within a community. Although both social cohesion and sense of community are inherently collective constructs, survey responses were collected at the individual level. As such, these outcomes reflect each respondent’s individual perceptions of their community rather than objective community-wide measures.

#### Sociodemographic variables and other covariates

Other variables included: sex, age, race/ethnicity, annual household income, employment status, education, use of other neighborhood parks, and CPI park site. Sex was analyzed as a dichotomous variable with levels of *“Male”* and *“Female.”* Age was analyzed as a categorical variable with three levels: *“18-34y”*,* “35-49y”* and *“50-89y”.* Race/ethnicity was categorized into four levels: “*Non-Latino/a White”*,* “Non-Latino/a Black”*,* “Latino/a”* and *“Other or multiracial*,*”* which included individuals who indicated they were Asian/Pacific Islander, American Indian/Alaskan Native or more than one race. Annual household income was captured as: *“Less than $25*,*000*,*” “$25*,*000-$75*,*000*,*” “$75*,*000-$150*,*000*,*”* and *“$150*,*000 or more.”* Employment status was captured as *“Employed or self-employed”* vs. *“Not employed”*, and education as “*One or more years of college”* vs. *“High school graduate or less”.* Frequency of use of other (non-CPI) neighborhood parks was recorded as: *“Not at all*,*” “< Once per week*,*” “Once per week*,*”* and *“> Once per week.”*

### Analysis

All descriptive and inferential analyses were done with the application of raked survey weights. Sociodemographic variables and other covariates were summarized using the weighted n (%) or mean (SD) and stratified by dichotomous study park use (< Once per week or ≥ Once per week). For continuous park use frequency, we summarized the mean (SD) number of park visits per month by levels of each sociodemographic variable. Outcome measures were also summarized as the mean (SD) SCT and SCI-2 scores by sociodemographic variables. Chi-squared tests for independence were performed using Rao and Scott’s second-order correction for complex survey-weighted designs across categorical variables and dichotomous park use [40]. Continuous measures were compared across groups with t-tests or ANOVA for normally distributed variables, and Wilcoxon rank-sum or Kruskal-Wallis tests for non-normally distributed variables, adapted for complex survey designs.

Linear regression models were fit to measure associations between park use frequency and the two outcomes: social cohesion and trust and sense of community. Six models were fit for each outcome to explore associations with different exposure definitions (i.e., continuous and dichotomous park use variables), covariate adjustments, and within different sociodemographic subgroups. Model specifications are explained in detail in Table 1. Due to the limited number of study sites (*n* = 8), we included study site as a fixed effect in adjusted models rather than employing a multilevel modeling or random effects approach. As supported by methodological literature, random-effects variance estimates can be unstable and less reliable with a small number of clusters; multilevel models are generally only recommended when more clusters are available [41].

**Table 1:** Linear regression model specifications

Model	Exposure Definition (CPI-Renovated Park Use)^a^	Covariates	Stratification^b^
Outcome: Social Cohesion and Trust (SCT Score) – construct measuring the degree to which neighbors perceive mutual trust, shared values, and a willingness to support one another in their community			
**A.1**	Continuous	None	None
**A.2**	Dichotomous	None	None
**B.1**	Continuous	Sex, age, race/ethnicity, annual household income, employment status, education, other park usage, and park site	None
**B.2**	Dichotomous	Sex, age, race/ethnicity, annual household income, employment status, education, other park usage, and park site	None
**C.1**	Continuous	Sex, age, race/ethnicity, annual household income, employment status, education, other park usage, and park site	Annual household income
**C.2**	Dichotomous	Sex, age, race/ethnicity, annual household income, employment status, education, other park usage, and park site	Annual household income, race/ethnicity
**Outcome: Sense of Community (SCI-2 Score) – multidimensional measure of an individuals’ psychological connection to a community**,** encompassing membership**,** influence**,** fulfillment of needs**,** and shared emotional connection**			
**D.1**	Continuous	None	None
**D.2**	Dichotomous	None	None
**E.1**	Continuous	Sex, age, race/ethnicity, annual household income, employment status, education, other park usage, and park site	None
**E.2**	Dichotomous	Sex, age, race/ethnicity, annual household income, employment status, education, other park usage, and park site	None
**F.1**	Continuous	Sex, age, race/ethnicity, annual household income, employment status, education, other park usage, and park site	Annual household income, race/ethnicity
**F.2**	Dichotomous	Sex, age, race/ethnicity, annual household income, employment status, education, other park usage, and park site	Annual household income, race/ethnicity

^a^ Continuous = days of CPI-renovated park use; dichotomous = level of CPI-renovated park use (< Once per week vs. ≥ Once per week)

^b^Interaction terms between park use variables and sex, race/ethnicity, and annual household income were explored, but only the models that resulted in a joint F-test p-value < 0.20 and at least one individual interaction coefficient with *p* < 0.05 were reported

In the results, we report beta estimates from the linear regression models with their corresponding 95% confidence intervals and p-values. For stratified models, interactions between park use variables and sex, race/ethnicity, and income were explored. Interaction terms were first screened using joint F-tests, with a threshold of *p* < 0.20. Models were reported only when the joint test met this criterion and at least one individual interaction coefficient was statistically significant at *p* < 0.05. Descriptive statistics were generated using the *gtsummary* package in R software v.4.5.1 (https://www.R-project.org). Linear regression models were fit using PROC SURVEYREG in SAS software v.9.4 (SAS Institute Inc., Cary, NC, USA). Assumptions for linear regression were verified using residual plots. Separate, unweighted regression models were fit using PROC REG to obtain variance inflation factors (VIFs) to confirm the absence of multicollinearity (VIF < 3) across variables in all models.

## Results

### Sample characteristics

The survey-weighted sample was predominantly female (53%) with an average age of 44.8 years (SD 17.8, Table 2). The sample was predominantly Latino/a (40%) and Non-Latino/a Black (28%), with Other or Multiracial individuals comprising 21% of the sample and Non-Latino/a Whites comprising 11% of the sample. The sample had a large proportion of low-income individuals where 30% reported an annual household income below $25,000 and another 38% reported income between $25,000-$75,000. Forty-three percent of the sample were not employed, which included those who were retired, unemployed, unable to work, students, or homemakers. Forty-five percent of the sample had a high school degree or less education. In the past 30 days, 42% of the sample reported visiting their CPI-renovated study park once per week or more, and 40% of the sample reported visiting other (non-CPI) neighborhood parks once per week or more. On average, study participants visited their CPI-renovated study park 5.8 times in the past 30 days (SD 8.7).

**Table 2:** Sample characteristics stratified by CPI-renovated park use

	Overall sample	Level of CPI-Renovated Park Use		
	*n *= 2000^a^	< Once per Week *n* = 1,159^a^	≥ Once per Week *n *= 841^a^	*p*-value^b^
**Sex**				
Female	1057 (53)	640 (55)	417 (50)	0.228
Male	943 (47)	519 (45)	424 (50)	
**Age**				
Mean (SD)	44.8 (17.8)	46.3 (18.1)	42.8 (17.1)	0.059
**Age Group**				
18-34y	646 (32)	343 (30)	303 (36)	0.027
35-49y	533 (27)	282 (24)	251 (30)	
50-89y	821 (41)	534 (46)	287 (34)	
**Race/Ethnicity**				
Latino/a	801 (40)	433 (37)	368 (44)	0.231
Non-Latino/a Black	558 (28)	354 (31)	205 (24)	
Other or multiracial	420 (21)	232 (20)	189 (22)	
Non-Latino/a White	220 (11)	140 (12)	79 (9)	
**Annual Household Income**				
Less than $25,000	604 (30)	320 (28)	284 (34)	0.213
$25,000 to $75,000	758 (38)	431 (37)	327 (39)	
$75,000 to $150,000	386 (19)	245 (21)	141 (17)	
$150,000 or More	252 (13)	162 (14)	90 (11)	
**Employment**				
Employed or self-employed	1136 (57)	654 (56)	483 (57)	0.829
Not employed	864 (43)	505 (44)	358 (43)	
**Education**				
One or more years of college	1104 (55)	724 (62)	380 (45)	< 0.001
High school graduate or less	896 (45)	435 (38)	461 (55)	
**Use of Other Neighborhood Parks**				
Not at all	633 (32)	449 (39)	184 (22)	< 0.001
< Once per week	569 (28)	331 (29)	238 (28)	
Once per week	515 (26)	222 (19)	293 (35)	
> Once per week	282 (14)	156 (13)	126 (15)	
**Days of CPI-Renovated Park Use**				
Mean (SD)	5.8 (8.7)	0.6 (0.9)	13.0 (9.5)	< 0.001

^a^Weighted n (%); mean (SD)

^b^Pearson’s Chi-squared test with Rao & Scott adjustment; Design-based Wilcoxon rank-sum test for complex survey samples

The prevalence of frequent (≥ once per week) CPI-renovated park use significantly differed by age group (*p* = 0.027) and education level (*p* < 0.001, Table 2). Individuals who used CPI-renovated parks ≥ once per week were younger (36% aged 18-34y and 30% aged 35-49y) and less educated (55% with high school degree or less) than those who used CPI-renovated parks < once per week (30% aged 18-34y, 24% 35-49y, and 38% with high school degree or less). Furthermore, individuals who frequently visited CPI renovated parks also tended to visit other neighborhood parks frequently (*p* < 0.001), with 50% of individuals who reported visiting the CPI-renovated park ≥ once per week also reporting visiting other neighborhood parks ≥ once per week.

Significant differences in average days of CPI-renovated park use also were observed by age (*p* = 0.004) and education level (*p* < 0.001, Table 3), with average number of visits decreasing as age and education level increased. Average SCT scores demonstrated a modest increasing trend with increasing income levels (*p* = 0.023) and marginally differed by race/ethnicity (*p* = 0.094), with non-Latino/a Black and White individuals exhibiting the highest perceived social cohesion and trust. No significant differences in SCI-2 scores were found across sociodemographic variables.

**Table 3:** Mean days of CPI-renovated park use in the past 30 days, social cohesion and trust scores, and sense of community index scores by sample demographic characteristics

	Days of CPI-Renovated Park Use		Social Cohesion and Trust (SCT) Score		Sense of Community Index (SCI-2) Score	
	Mean (SD)	*p*-value^a^	Mean (SD)	*p*-value^b^	Mean (SD)	*p*-value^b^
Sex						
Female	5.73 (8.81)	0.143	3.20 (0.47)	0.597	52.8 (15.9)	0.318
Male	5.93 (8.56)		3.22 (0.49)		54.1 (14.6)	
**Age Group**						
18-34y	7.02 (9.40)	0.004	3.21 (0.45)	0.517	54.1 (15.6)	0.655
35-49y	5.77 (7.95)		3.24 (0.49)		52.8 (15.2)	
50-89y	4.91 (8.47)		3.18 (0.50)		53.2 (15.1)	
**Race/Ethnicity**						
Latino/a	6.38 (9.11)	0.226	3.16 (0.52)	0.094	52.6 (15.9)	0.649
Non-Latino/a Black	5.47 (8.85)		3.26 (0.47)		54.4 (15.8)	
Other or multiracial	5.65 (8.11)		3.18 (0.37)		54.0 (13.6)	
Non-Latino/a White	5.00 (7.68)		3.30 (0.53)		52.9 (14.8)	
**Annual Household Income**						
Less than $25,000	6.67 (9.78)	0.602	3.18 (0.52)	0.023	53.3 (15.8)	0.933
$25,000 to $75,000	5.74 (8.40)		3.18 (0.42)		53.1 (14.1)	
$75,000 to $150,000	4.86 (7.29)		3.22 (0.56)		53.6 (17.4)	
$150,000 or More	5.50 (8.62)		3.33 (0.44)		54.2 (14.0)	
**Employment**						
Employed or self-employed	5.54 (8.04)	0.810	3.20 (0.50)	0.849	52.5 (15.4)	0.107
Not employed	6.20 (9.48)		3.21 (0.47)		54.6 (15.0)	
**Education**						
One or more years of college	5.02 (8.32)	< 0.001	3.21 (0.50)	0.963	52.6 (15.3)	0.171
High school graduate or less	6.81 (9.05)		3.21 (0.46)		54.4 (15.2)	

^a^Design-based Wilcoxon rank-sum test or Kruskall-Wallis test for complex survey samples

^b^Design-bassed t-test or ANOVA for complex survey sample

### Associations between CPI-renovated park use and social cohesion and trust

With continuous park use as the exposure, significant positive associations were identified between number of past-month visits to CPI-renovated study parks and SCT scores in both crude and adjusted models (A.1 and B.1, Table 4). In the adjusted model, each additional visit to a CPI-renovated study park within the past 30 days was associated with a 0.012-point increase in SCT score (95% CI: 0.008–0.016; *p* < 0.001) on a 1-5-point scale. When examining dichotomous park use frequency as the exposure, significant associations with SCT scores were also observed in both crude and adjusted models (A.2 and B.2, Table 4), with the adjusted models indicating that individuals who visited CPI-renovated parks ≥ once per week had SCT scores that were 0.179 points higher on average (95% CI: 0.102–0.255) than those who visited < once per week (*p* < 0.001).

**Table 4:** Associations between CPI-renovated park use frequency and social cohesion and trust (SCT)

	Days of CPI-Renovated Park Use				Level of CPI-Renovated Park Use (< Once per Week vs. ≥ Once per Week)^a^	
	Beta (95% CI)^b^	*p*-value^b^			Beta (95% CI)^b^	*p*-value^b^
**Model A.1**				**Model A.2**		
Crude	0.012 (0.008, 0.017)	< 0.001		Crude	0.175 (0.097, 0.253)	< 0.001
**Model B.1**				**Model B.2**		
Adjusted	0.012 (0.008, 0.016)	< 0.001		Adjusted	0.179 (0.102, 0.255)	< 0.001
**Model C.1**				**Model C.2**		
				Race/Ethnicity		
				Latino/a	0.199 (0.069, 0.328)	0.003
				Non-Latino/a Black	0.208 (0.062, 0.354)	0.005
				Other or multiracial	-0.011 (-0.161, 0.139)	0.883
				Non-Latino/a White	0.213 (0.027, 0.399)	0.025
				Joint F-Test p-value		0.093
Annual Household Income				Annual Household Income		
Less than 25,000	0.010 (0.003, 0.017)		0.007	Less than 25,000	0.222 (0.043, 0.400)	0.015
$25,000 to $75,000	0.013 (0.007, 0.019)		< 0.001	$25,000 to $75,000	0.150 (0.044, 0.256)	0.005
$75,000 to $150,000	0.024 (0.013, 0.035)		< 0.001	$75,000 to $150,000	0.286 (0.125, 0.448)	< 0.001
$150,000 or More	0.006 (-0.005, 0.017)		0.289	$150,000 or More	-0.050 (-0.229, 0.130)	0.587
Joint F-Test p-value			0.101	Joint F-Test p-value		0.047

^a^ Reference category for level of park use: < once per week

^b^ Estimated from linear regression models with SCT score as the outcome variable. Models A.1-A.2 included the CPI-renovated park use exposure alone and Models B.1-B.2 included the CPI-renovated park use exposure plus additional adjustments for sex, age, race/ethnicity, annual household income, employment status, education, other park usage, and park site. Models C.1-C.2 show stratified results and included the same covariates as Models B.1-B.2, in addition to interaction terms between CPI-renovated park use and race/ethnicity and annual household income

Evidence of interaction was found between continuous park use frequency and annual household income (Model C.1), as well as between dichotomous park use frequency with both annual household income and race/ethnicity (Model C.2), as shown in Table 4. Stratified analyses revealed that individuals with upper-middle income levels ($75,000 to $150,000) had the strongest association between CPI-renovated park use frequency and SCT, with a 0.024-point increase in SCT for each additional monthly visit to a study park (95% CI: 0.013–0.035; *p* < 0.001). Those with annual household incomes above $150,000 showed no relationship between SCT and park use frequency in either Model C.1 or C.2. In Model C.2, similar associations between dichotomous levels of park use frequency and SCT were observed among Latino/as, non-Latino/a Blacks, and non-Latino/a Whites, while no association was observed among the other/multiracial group.

### Associations between CPI-renovated park use and sense of community

With continuous park use as the exposure, significant positive associations were identified between number of past-month visits to CPI-renovated study parks and SCI-2 scores in both crude and adjusted models (D.1 and E.1, Table 5). In the adjusted model, each additional visit to a CPI-renovated study park within the past 30 days was associated with a 0.464-point increase in SCI-2 score (95% CI: 0.335–0.593; *p* < 0.001) on a 0-72-point scale. When examining dichotomous park use frequency as the exposure, significant associations with SCT scores were also observed in both crude and adjusted models (D.2 and E.2, Table 5), with the adjusted models indicating that individuals who visited CPI-renovated parks ≥ once per week had SCI-2 scores that were 6.06 points higher on average (95% CI: 3.73–8.38) than those visiting < once per week (*p* < 0.001).

**Table 5:** Associations between CPI-renovated park use frequency and sense of community (SCI-2)

	Days of CPI-Renovated Park Use			Level of CPI-Renovated Park Use (< Once per Week vs. ≥ Once per Week)^a^	
	Beta (95% CI)^b^	*p*-value^b^		Beta (95% CI)^b^	*p*-value^b^
**Model D.1**			**Model D.1**		
Crude	0.507 (0.352, 0.662)	< 0.001	Crude	7.17 (4.75, 9.60)	< 0.001
**Model E.1**			**Model E.2**		
Adjusted	0.464 (0.335, 0.593)	< 0.001	Adjusted	6.06 (3.73, 8.38)	< 0.001
**Model F.1**			**Model F.2**		
Race/Ethnicity			Race/Ethnicity		
Latino/a	0.676 (0.480, 0.871)	< 0.001	Latino/a	7.51 (3.78, 11.24)	< 0.001
Non-Latino/a Black	0.478 (0.243, 0.714)	< 0.001	Non-Latino/a Black	8.28 (4.07, 12.49)	< 0.001
Other or multiracial	0.307 (-0.002, 0.615)	0.051	Other or multiracial	1.29 (-4.08, 6.67)	0.638
Non-Latino/a White	0.338 (0.102, 0.573)	0.005	Non-Latino/a White	5.17 (0.85, 9.49)	0.019
Joint F-Test p-value		0.087	Joint F-Test p-value		0.178
Annual Household Income			Annual Household Income		
Less than 25,000	0.296 (0.060, 0.532)	0.014	Less than 25,000	3.76 (-0.65, 8.18)	0.095
$25,000 to $75,000	0.348 (0.173, 0.523)	< 0.001	$25,000 to $75,000	5.38 (1.97, 8.80)	0.002
$75,000 to $150,000	0.854 (0.491,1.217)	< 0.001	$75,000 to $150,000	10.41 (5.30, 15.51)	< 0.001
$150,000 or More	0.300 (0.075, 0.526)	0.009	$150,000 or More	2.70 (-2.28, 7.69)	0.288
Joint F-Test p-value		0.056	Joint F-Test p-value		0.142

^a^ Reference category for levels of park use: < once per week

^b^ Estimated from linear regression models with SCI-2 score as the outcome variable. Models D.1-D.2 included the CPI-renovated park use exposure alone and Models E.1-E.2 included the CPI-renovated park use exposure plus additional adjustments for sex, age, race/ethnicity, annual household income, employment status, education, other park usage, and park site. Models F.1-F.2 show stratified results and included the same covariates as Models E.1-E.2, in addition to interaction terms between CPI-renovated park use and race/ethnicity and annual household income

Evidence of interaction was found between continuous park use frequency with both race/ethnicity and annual household income (Model F.1), as well as between dichotomous park use frequency with both race/ethnicity and annual household income (Model F.2), as shown in Table 5. Latino/a and Non-Latino/a Black participants showed the strongest associations between SCI-2 scores and measures of park use frequency. For Latino/as, each additional day of CPI-renovated park use in the past month was associated with a 0.676-point increase (95% CI: 0.480–0.871; *p* < 0.001) in SCI-2 score. For non-Latino/a Blacks, the association was 0.478 additional SCI-2 points for every additional past-month visit (95% CI 0.243–0.714; *p* < 0.001). Similarly, across income groups, associations between park use frequency measures and SCI-2 scores were the strongest among middle-income individuals with an annual household income between $75,000 and $150,000, who showed an association of 0.854 additional SCI-2 points for every additional past-month CPI-renovated park visit (95% CI: 0.491–1.217; *p* < 0.001). The lowest (<$25,000) and highest (>$150,000) income groups showed weak associations between days of study park use and SCI-2 score and no significant associations in the model with dichotomous park use as the exposure.

## Discussion

In the context of eight recently renovated neighborhood parks situated within low-income communities in NYC, park use frequency was positively associated with individual perceptions of social cohesion and trust and sense of community. These associations were significant across both continuous days and dichotomous levels (< once per week vs. ≥ once per week) of renovated park use. We also found evidence of differential associations by race/ethnicity and income, with the strongest associations present among Latino/as, Non-Latino/a Blacks, and middle-income individuals (with annual household income $75,000 to $150,000).

Our primary findings align with existing literature suggesting that urban green spaces represent an important feature of the urban built environment associated with community social cohesion [4, 13, 42–44]. Studies have shown that among low income urban communities in particular, individuals who reside within one kilometer of a green space may perceive less of a shortage of social support [44]. Moreover, park quality may be an important precondition to the link between park use and community social cohesion. Low quality parks that are perceived as dangerous have the potential to threaten perceptions of social cohesion [26]. In contrast, high-quality urban green spaces with well-maintained amenities provide clean, safe, and comfortable settings for social gatherings with loved ones and community members. Frequent social interactions in public urban parks can foster a sense of connectedness and enhance social ties [13], which may boost one’s perception of community social cohesion. Well-maintained parks may also be an indicator for neighborhood upkeep, which is associated with increased levels of perceived safety and social capital [45, 46]. It is important to note that this relationship is likely bidirectional, as existing social cohesion may drive local efforts to maintain or improve park quality through community organizing or volunteerism. Additional studies have found that park design features such as amenities and physical activity facilities are also positively associated with neighborhood social cohesion [43].

Building on this evidence, one plausible mechanism linking social cohesion to individual-level health outcomes is the accumulation of social capital through neighborhood interactions in shared public spaces. Parks, particularly those that are safe and well-maintained, can serve as relational infrastructure that fosters community connection and mutual support [12, 47, 48]. For example, community-oriented park activities such as family gatherings, children’s playdates, and neighborhood cultural events may facilitate the informal sharing of local resources, emotional support, and health-related information. Consequently, a growing body of empirical research suggests that social cohesion may play a powerful mediating role in the relationship between green spaces and health outcomes, particularly in relation to general health and mental well-being [44, 47, 49, 50].

Despite no significant differences in park use frequency by race/ethnicity, Non-Latino/a Black and Latino/a individuals showed strong associations between renovated park use and sense of community. Cultural differences in park use behaviors may offer one explanation for these observed differences. Prior studies have shown that people of color tend to use parks for social gatherings more than White individuals [23]. For example, one study assessing park use activities across 50 parks in Los Angeles found that compared to White residents, Spanish-speaking Latino/a residents were more likely to engage in social interactions in the parks [51]. Similarly, a qualitative study of Latina women in the Northeastern U.S. found that foreign-born Latinas perceived community parks as a setting for their families to socialize with other families of similar cultural backgrounds [52]. Like Latino/as, Black individuals are more likely than their White counterparts to visit parks for a gathering with friends and family according to a national survey [53]. If minoritized residents use community parks for social activities more frequently than White residents, it is plausible that the impact of park use on perceptions of social cohesion may also be greater in these groups compared to White residents.

In our study, individuals earning between $75,000-$150,000 annually reported significantly fewer days in the park per month than both lower-income (<$25,000) and higher-income (≥$150,000) residents, yet they showed the strongest associations between park use and perceived social cohesion. One possible explanation is that middle-income individuals may engage in more community-based social activities outside of parks compared to their lower- or higher-income counterparts, potentially boosting their overall perception of social cohesion, and translating to greater social engagement when they do visit parks. Pew Research Center data show that two-thirds of individuals earning at least $75,000 annually belong to at least one community group, compared to fewer than half of those earning under $30,000 [54]. In contrast, lower-income individuals often face barriers to community engagement due to time poverty and economic precarity, with many juggling multiple jobs or caregiving responsibilities that leave limited time for social activities and civic participation [55]. At the other end of the spectrum, higher-income individuals earning over $150,000 annually also showed little-to-no association between park use and perceived social cohesion. Nationally representative studies spanning multiple decades suggest that higher-income individuals tend to spend less time socializing with neighbors and more time socializing with friends [56]. Additional studies have found that parks may be a less important factor in fostering social contacts for people with a higher socioeconomic status [44]. With more disposable income, higher-income individuals may prefer commercial venues (e.g., restaurants, gyms) or private settings for socializing, reducing their reliance on public parks as sites of community connection. Taken together, these observed patterns suggest that the social value of parks is likely shaped by the broader contexts in which different income groups seek community, with middle-income individuals potentially positioned to benefit most from the opportunities for social connections that parks can provide.

Our findings must also be situated within the unique economic context of NYC: although the median annual household income of New Yorkers was $76,000 in 2023 [57], given the city’s high cost of living, estimates suggest that a single adult may require over $130,000 to live comfortably, while a family of four may need upwards of $300,000 [58]. As a result, many residents in the $75,000-$150,000 bracket, despite being above the statistical median, may still experience financial strain and heightened awareness of their relative socioeconomic position. Relative deprivation theory offers one lens for interpreting the experience of this “squeezed middle”, as individuals may perceive themselves as disadvantaged compared to wealthier peers in the city, while simultaneously experiencing economic and social advantages over lower-income groups. This socioeconomic positioning may generate feelings of insecurity and relative deprivation [59, 60], which in turn may increase the psychological value of contexts that foster community belonging, such as visiting neighborhood parks, even if visits are relatively infrequent. Even so, this is only one possible lens through which to interpret our findings, and further research is needed to clarify the potential role of relative deprivation in the differential social benefits of parks by income level.

### Policy implications

Our findings support the positioning of urban parks as critical public health infrastructure with the potential to advance health equity goals in diverse, underserved urban areas. Importantly, equity-focused park investments modeled on the CPI should be accompanied by increased and sustained funding for maintenance and programming, ensuring that the public health benefits of renovated parks extend beyond initial capital projects. Our differential findings by race/ethnicity highlight the importance of tailored community engagement models for Black and Latino/a residents, who in our study exhibited the strongest connection between renovated park use and sense of community compared to other racial/ethnic groups. Notably, the SPARCS project, from which these data were drawn, represents one such community-engaged intervention model, designed to activate renovated CPI parks through culturally tailored programming that reflects neighborhood needs and preferences [27]. At the same time, low-income residents, who reported more frequent park use but weaker associations with social cohesion, may require complementary supports, such as programming that reduces barriers to participation in park-based social activities. Finally, to ensure park renovation and programming investments achieve public health and health equity goals, policymakers should incorporate social metrics into evaluation frameworks, including repeated assessments of social cohesion measures across diverse, population-representative neighborhood samples.

### Limitations and strengths

A limitation of this study is its cross-sectional design; thus, no causality could be inferred, and reverse causation may be a possibility. For example, rather than more frequent park use leading to higher perceived social cohesion, individuals who already feel a strong sense of community cohesion may be more inclined to engage in community-based activities, such as socializing or volunteering in public spaces. Future studies should adopt longitudinal and quasi-experimental designs to appropriately establish temporality of park use and changes in perceived social cohesion. Another limitation is the use of self-reported park use frequency in the past month, which is subject to recall and social desirability biases and may not accurately capture all park use. Future research should incorporate more objective and continuous measures of park use, using emerging tools for assessing population mobility patterns, such as geolocation data from GPS-enabled smartphones and smartwatches [61]. Furthermore, although we adjusted for relevant individual-level sociodemographic confounders and park site, additional contextual confounders and effect modifiers may also influence the relationship between renovated park use and perceived community social cohesion. Future research could evaluate these associations in the presence of contextual modifiers, including neighborhood characteristics (e.g., traffic safety, crime rates, gentrification) and park characteristics (e.g., night-time lighting, maintenance, accessibility).

Our study was strengthened by the random, population-representative sample and triangulation of findings across two validated, complementary quantitative measures of perceived community social cohesion (SCT and SCI-2). Though we could not control for neighborhood conditions that may precede or follow improvements in park quality, such as an increase in policing or sanitation services, we did control for park quality itself by focusing only on recently renovated park sites. This represents a unique contribution to the literature, as heterogeneity in park quality often confounds relationships between park use and health outcomes. In addition, the large sample of predominantly low-to-middle income, minoritized residents allowed us to explore unique relationships stratified by race/ethnicity and income levels, which also represents an important contribution to the literature.

## Conclusion

In a large, population-representative sample of NYC residents living near recently renovated parks, we found significant positive associations between frequency of use of the renovated parks and two measures of perceived neighborhood social cohesion: social cohesion and trust and sense of community. We observed the strongest associations among Latino/a, Black, and middle-income participants with annual household earnings of $75,000-$150,000 per year. These findings emphasize the potential role of high-quality urban parks in supporting community well-being through frequent use of renovated park spaces.

## Data Availability

The data that support the findings of this study are available from the authors upon reasonable request.
